# Physiochemical Characteristics of Hot and Cold Brew Coffee Chemistry: The Effects of Roast Level and Brewing Temperature on Compound Extraction

**DOI:** 10.3390/foods9070902

**Published:** 2020-07-09

**Authors:** Niny Z. Rao, Megan Fuller, Meghan D. Grim

**Affiliations:** Department of Biological and Chemical Sciences, College of Life Sciences, Thomas Jefferson University, East Falls Campus, Philadelphia, PA 19144, USA; megan.fuller@jefferson.edu (M.F.); Meghan.Grim@jefferson.edu (M.D.G.)

**Keywords:** coffee, cold brew, acidity, antioxidant, coffee roasting

## Abstract

The role of roasting in cold brew coffee chemistry is poorly understood. The brewing temperature influences extraction processes and may have varying effects across the roast spectrum. To understand the relationship between brew temperature and roast temperature, hot and cold brew coffees were prepared from Arabica Columbian coffee beans roasted to light, medium, and dark levels. Chemical and physical parameters were measured to investigate the relationships among degree of roast, water temperature, and key characteristics of resulting coffees. Cold brew coffees showed differential extraction marked by decreased acidity, lower concentration of browned compounds, and fewer TDS indicating that cold water brewing extracts some compounds less effectively than hot water brewing. Compounds in coffee did exhibit sensitivity to degree of roast, with darker roasts resulting in decreased concentrations for both hot and cold brew coffees. Total antioxidant capacity (TAC) was only sensitive to degree of roast in cold brew coffees, while hot brew coffees had a constant TAC for all three roast levels. This indicates that the solid bean matrix and its chemical constituents interact with cold water differently than with hot water. Surface wetting, pore dynamics, and solubility all contribute to the extraction potential during brewing and are all functions of water temperature.

## 1. Introduction

Coffee chemistry is determined by numerous factors, including the varietal of bean, region of origin, growing conditions, roasting process, grind size distribution, water chemistry, and temperature of water used during extraction. Cold brewing has become a popular coffee production method, where coffee is prepared at room temperature (20 to 25 °C or colder) and steeped for 8 to 24 h—much longer than the time required by traditional hot brewing methods. While there exists a significant body of literature on the relationship between coffee bean roasting and hot brew coffee chemistry [1,2,3], there is little understanding of how cold brew coffee chemistry is affected by bean roast.

Green coffee beans must be roasted in order to produce the complex suite of bioactive compounds necessary for a flavorful and aromatic cup of coffee. During roasting, the solid matrix of the coffee bean undergoes important physical and chemical alterations as a function of internal bean temperature. Roasting is achieved by applying hot air to the beans such that a minimum internal bean temperature of 190 °C is sustained for some length of time, usually no less than 3 min [1]. 

Due to the generation and release of gases during heating, the roasting process results in lower bean density and greater bean volume [2]. During this expansion and off-gassing, significant chemical synthesis and degradation occur within the solid bean matrix. The application of heat to green coffee beans results in substantial mass loss (12 to 18%) and pyrolysis of organic components [3]. At bean temperatures above 180 °C, exothermic reactions of polysaccharides, proteins, and phenolic acids produce compounds that give coffees their distinct flavors. Natural phenolic and other acids are lost during heating, while Maillard reaction products, including melanoidins, are generated [4]. Melanoidin compounds are responsible for the brown color of roasted beans and are known to contribute to the antioxidant activity of coffee [5]. The physical and chemical alterations that occur in the bean matrix during roasting are known to change not only the acidity of the resulting coffee, but also the antioxidant activity of the final beverage [6].

With the popularization of cold brew coffee, there is an interest in understanding the relationship between cold brew coffee chemistry and degree of bean roast. Extraction during cold brewing is characterized by low-energy, prolonged water–grind contact times. Early research on cold brew coffee chemistry found that grind size and roast [7,8], brewing method [9], and region of origin [10] all influence the bioactive compounds found in cold brew coffee. This nascent body of research has shown that hot and cold brew coffees differ in relatively small but important ways, particularly in the total antioxidant capacity (TAC) of the resulting coffees. 

This study aims to understand the interaction between aqueous extraction and degree of roast for a single-origin coffee from Columbia as a function of water temperature. It is known that degree of roast significantly affects the chemistry of hot brew coffee [6,11,12], but little is known about the effect of degree of roast on cold brew coffee chemistry. This work will measure pH, total titratable acidity (TTA) at pH 6.5, total dissolved solids (TDS), browned compounds (Abs 420 nm), caffeine concentration, total caffeoylquinic acid (CQA) concentration (the sum of three caffeoylquinic acid isomers), and TAC of both hot and cold brew coffee prepared from light, medium, and dark roast beans. This work will also investigate the differential extraction of key compounds for hot and cold brewing methods at three roast levels to better understand the role of water temperature in facilitating the solubility and extraction of soluble solids. 

## 2. Materials and Methods

### 2.1. Coffee Bean Preparation

Green, unroasted organic Columbian coffee beans were purchased from Golden Valley Coffee Roasters (West Chester, PA, USA). Samples of green coffee beans (250 g) were roasted in a bench top coffee roaster (Model No. KN-8828B-2K, HotTop, Hottop Coffee Roaster, Cranston, RI, USA) using the manufacturer’s default temperature-time setting. Beans were ejected from the roaster at three different final temperatures: 194 °C, 203 °C, and 209 °C, corresponding to R1, R2, and R3, respectively. A total of four batches of each roast were produced. The batches were mixed and frozen for a minimum of 12 h [13] and ground using a conical burr grinder (Model No. 560.01, Capresso, Montvale, NJ, USA) at the highest coarseness setting as designated by the manufacturer. The grinds were then sieved to retain particles between 500 µm and 2000 µm. ASTM sieves with mesh sizes #10 (2000 µm mesh opening) and #35 (500 µm mesh opening) were used. Sieved grinds were stored at −5 °C prior to brewing.

### 2.2. Reagents

Standards of 5-Caffeoylquinic acid (5-CQA, CAS: 327-97-9), 4-CQA (CAS: 905-99-7), and 3-CQA (CAS: 906-33-2), Caffeine (CAS: 58-08-2), Phosphoric acid (85% wt.) (CAS: 7664-38-2), Trolox (6-hydroxy-2,5,7,8-tetramethylchroman-2-carboxylic acid) (CAS: 53188-07-1), ethanol (CAS: 64-17-5), potassium persulfate (CAS: 7727-21-1), ABTS˙^+^ (2,2′-azionbis(3-ethylbenzothiazoline-6-sulfonic acid) diammonium salt (CAS: 30931-67-0), and Standardized 0.1 N NaOH (CAS: 1310-73-2) were purchased from Sigma-Aldrich (Milwaukee, WI, USA). HPLC-grade methanol (CAS: 67-56-1) was obtained from Fisher Scientific (Nazareth, PA, USA). Standard stock solutions of 2.5 mM Trolox were prepared in ethanol weekly. ABTS˙^+^ radical cation solutions were prepared every 48 h and stored in the dark at room temperature. DI water was used to brew the coffees.

### 2.3. Coffee Brewing and Storage 

Cold brew coffee was prepared by placing a sample of 20 g of coffee grinds with 200 mL DI water at room temperature in a beaker fitted with a French press plunger for 7 h, after which the coffee solution was pressed by depressing the plunger. 

Hot brew coffee was prepared by pouring 200 mL DI water at 100 °C over 20 g of coffee grinds in a beaker fitted with a French press plunger. After 6 min, the coffee was pressed by depressing the plunger.

All brewed coffee was filtered using a 0.45 µm Acrodisc^®^ Syringe Filter with a Universal PTFE Membrane (Pall, New York, NY, USA) [14]. All coffee samples were analyzed within 10 min of brewing.

### 2.4. HPLC Analysis

Standard solutions and coffee extracts were analyzed using a methodology adapted from GL Sciences Technical Note No. 67 [15]. All analyses were performed at 25 °C with a mobile phase mixture of 75% mobile phase A and 25% mobile phase B (A: 95% 2.0 mM phosphoric acid and 5% methanol; B: 95% methanol and 5% 2.0 mM phosphoric acid) on an Agilent 1200 Series high-performance liquid chromatography (HPLC) system was fitted with a Supelco C-18, 5 µm column (15 cm × 4.6 cm) (Supelco, Bellefonte, PA), and a C-18 guard column. The mobile phase flow rate was set at 1.0 mL/min with an injection volume of 10.0 µL. CQA isomers and caffeine were detected using a diode array detector at 325 nm and 280 nm, respectively. Concentrations of 5-CQA and caffeine were quantified via standard calibration curves. Retention time of 3-CQA and 4-CQA isomers was determined using standard solutions and quantitation of these two isomers was accomplished using the area of the 5-CQA standard combined with the respective molar extinction coefficients of the other two isomers as reported previously [16,17,18]. Three batches of coffee were brewed for each roast level, and each batch of brewed coffee was analyzed in triplicate (*n* = 9).

### 2.5. pH and Total Titratable Acidity Measurement

The pH of each brewed coffee sample was measured with a Mettler Toledo FiveEasy^TM^ F20 benchtop pH/mV meter. A 40 mL aliquot of coffee brew was titrated with 0.1 N NaOH to a pH of 6.5 [8] to determine the TTA of each coffee. Three batches of coffee were brewed for each roast and each batch was analyzed once (*n* = 3).

### 2.6. Total Antioxidant Capacity (TAC) Measurements

The TACs of hot and cold brew coffees were determined using an ABTS radical cation decolorization assay modified from Vignoli et al. [6] and Re et al. [19]. To summarize the procedure, a stock solution of ABTS˙^+^ radical cation was made by mixing equal parts 7.0 mM ABTS and 2.45 mM potassium persulfate. The mixture was allowed to develop in the dark at 20 °C for up to 16 h to reach optimal absorbance at 734 nm. The ABTS˙^+^ stock solution was then diluted with DI water such that the absorbance of the solution was between 0.80 and 0.90 at 734 nm. The TAC of Trolox standards at 734 nm were determined by mixing 30 µL of 2.5 mM Trolox solution with 3.0 mL of diluted ABTS˙^+^ solution. The absorbance was then measured exactly 6 min after mixing using a Thermo Scientific Evolution 201 spectrophotometer. The ABTS˙^+^ scavenging capacity was determined by the absorbance difference between the ABTS˙^+^ solution and the Trolox-ABTS˙^+^ sample.

The TAC of each coffee sample was determined by mixing 5.0 µL aliquot of filtered coffee with 3.0 mL of the dilute ABTS˙+ solution and the resulting solution was analyzed following the same analytical procedure for the Trolox standards. The TAC of each coffee sample was expressed as mmol Trolox equivalent per liter of brewed coffee. Three batches of coffee were brewed for each roast and each batch was analyzed in triplicate (*n* = 9).

### 2.7. Browned Compounds (Abs 420 nm)

The quantity of browned compounds generated during brewing has been used as an indicator for degree of caramelization and development of Maillard reactions, and was determined via the absorbance of diluted brewed coffee at 420 nm [20]. The brewed coffee was diluted 1:20 (*v*:*v*) with DI water and the absorbance of the solution at 420 nm was measured using a Thermo Scientific Evolution 201 spectrophotometer [21]. Three batches of coffee were brewed for each roast and each batch was analyzed in duplicate (*n* = 6).

### 2.8. Total Dissolved Solids

The TDS in both hot and cold brew coffees were measured following the protocol outlined by Moreno et al. [22]. The °Brix of each coffee brew was measured using a Fisherbrand™ Handheld Digital Brix/RI Refractometer (Fisher Scientific, Waltham, MA, USA), then converted to TDS (X_s_) as follows: X_S_ = 0.0087 × °Brix.

### 2.9. Statistical Analysis

Two-way ANOVA analysis with Tukey’s Honest Significant Difference (HSD) test was performed using an R script by Wessa [23]. Differences between means were considered significant at *p* < 0.05. All *F* values from the ANOVA analysis are included in the Supplementary Materials in Tables S1–S10.

## 3. Results and Discussion

Hot and cold brew coffees were prepared from Columbian beans roasted at three different degrees of roast: R1, R2, and R3, with R1 being the lightest and R3 being the darkest. The chemistry of each coffee was characterized by pH, TTA titrated to pH 6.5, TDS, browned compounds (Abs 420 nm), caffeine concentration, total CQA concentration (the sum of three caffeoylquinic acid isomers), and TAC as shown in Table 1. 

Hot and cold brew coffees were analyzed to understand the effect of bean roast level on coffee chemistry as a function of water extraction temperature. Water temperature affects compound solubility, and each of the three roast levels saw differential outcomes at varying extraction temperatures.

### 3.1. Acidity

**Figure 1:**
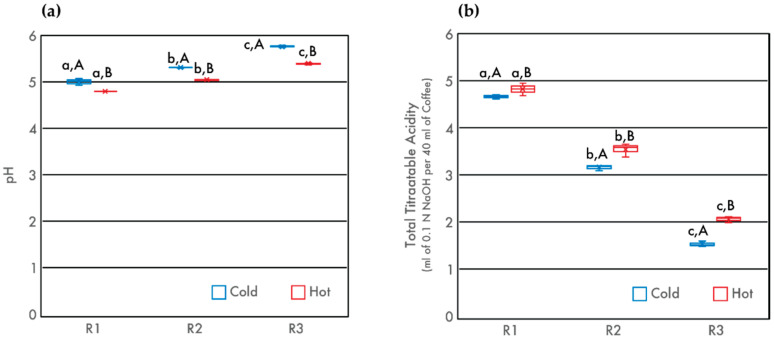
pH (**a**) and total titratable acids (TTA) (**b**) of cold and hot brew coffee samples as a function of degree of roast. Letters *a–c* denote significant (*p* < 0.05) differences among degrees of roast within the same brewing method as determined by the Tukey HDS post-tests. Letters *A* and *B* denote significant differences between cold and hot brewing methods at the same degree of roast.

The acidity of all coffee beverages decreased as degree of roast increased, in agreement with previous studies [24,25]. Cold brew coffees from each of the three roasts were found to be less acidic than their hot brew counterparts, with mean values differing by 0.20, 0.26, and 0.34 pH units for R1, R2, and R3, respectively (Table 1). These results support work by Cordoba et al., who found that cold brew coffees had statistically higher pH values than their hot brew counterparts for two varietals of Arabica beans, but these differences between hot and cold brews did not exceed 0.5 pH units [8]. As degree of roast increased, the TTA of all coffees decreased (Figure 1). There is an apparent loss of soluble protonated acidic compounds during the roasting process, due to either decomposition during pyrolysis or consumption in synthesis reactions [11]. For both pH and TTA, the differences between hot and cold brew coffees increased as a function of degree of roast, indicating that roast has more influence on the acidity of the final brew than that of the water extraction temperature. While these differences are relatively small, they illustrate that hot water extraction does yield increased availability and solubility of some acidic compounds. 

These results support past research on the relationship between roasting and pH in hot brew coffees. Moon et al. roasted and analyzed beans from various coffee producing regions including Nicaragua, Ethiopia, Panama, and Sumatra. From green beans to beans that were roasted to a maximum temperature of 250 °C, they found that pH increased with roast level across all regions [25]. Work by Ginz et al. found that coffee pH is, in large part, due to the formation of aliphatic acids during roasting, a mechanism that is dependent on precursor compounds such as sucrose [11]. The current study shows increased variability in TTA with increasing degree of roast. This suggests that physical and chemical changes in the coffee beans create a differential extraction process for cold and hot brew mass transfer across the solid bean matrix.

### 3.2. Total Dissolved Solids (TDS)

TDS increased with degree of roast for both hot brew and cold brew samples (Table 1). As was found in previous studies [8,26], TDS was higher for hot brew coffees than cold brew coffees at all roast levels. This difference in TDS between hot and cold brew coffees was enhanced as degree of roast increased. Although the decomposition of compounds during the roasting process may lead to a loss in soluble solids, new compounds are also formed during pyrolysis. In addition, the intense heat of roasting also damages the cellular matrix, which in turn makes compounds more readily extractable [6,27,28,29,30,31]. The difference in TDS between hot and cold brewing methods implies that the extraction of certain compounds is limited by the extraction temperature, due to the low solubility of these compounds in cold water.

### 3.3. Browned Compounds, CQA Concentration, and Total Antioxidant Capacity (TAC)

**Figure 2:**
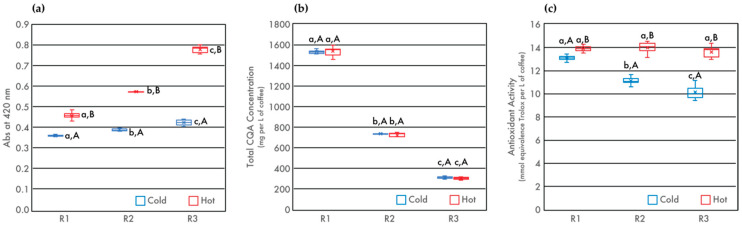
Absorbance at 420 nm (**a**), total CQA concentration (**b**), and total antioxidant capacity (TAC) (**c**) of cold and hot brew coffee as a function of degree of roast. Letters *a–c* denote significant (*p* < 0.05) differences among degrees of roast within the same brewing method as determined by the Tukey HDS post-tests. Letters *A* and *B* denote significant differences between cold and hot brewing methods at the same degree of roast.

The concentration of browned compounds in coffee can be characterized by absorbance at 420 nm. The browning of coffee beans during roasting is largely due to the production of melanoidin compounds [32,33]. Melanoidins are high-molecular weight products of the Maillard reaction and are known to contribute to a coffee’s brown color, flavor, and antioxidant capacity [5,21,34,35,36,37,38]. Consistent with previous studies [21,39,40,41], absorbance at 420 nm increased with degree of roast (Figure 2), suggesting an increase in the concentration of browned compounds. Similar to TDS, the difference in the concentration of browned compounds between cold and hot brew samples increased with degree of roast. The observed difference between hot and cold brew coffees was likely due to the formation of compounds during the roasting process that were not soluble in cold water. Hot brew coffees always had significantly more browned compounds than their cold brew counterparts, with browned compounds increasing as a function of roast level [21,30,39]. These complex melanoidin compounds are more available for extraction in hot water, likely due to temperature-dependent solubility effects and improved wetting. 

The total CQA concentration, as well as individual concentrations of 3-CQA, 4-CQA, and 5-CQA, are shown in Table 2. It is well established that, as roasting temperature increases, CQA concentrations in the solid bean matrix decrease due to compound degradation [6,25,42,43]. This current study found that, as degree or roast increased, the total CQA concentrations in both cold and hot brew coffees decreased. In addition, water extraction temperature did not cause differential extraction of CQA isomers. These results support earlier work by Fuller and Rao [7], who found that both hot and cold brew coffees prepared from Kona coffee beans showed no significant differences in CQA concentrations for the two roasts analyzed [7].

TAC for each of the hot and cold brew coffees prepared from the three roasts was determined using ABTS decolorization assays and is illustrated in Figure 2. Degree of roast did not significantly affect TAC in hot brew coffees, as shown by a two-way ANOVA analysis. For all roasts, hot brew coffee contained higher levels of TAC when compared to cold brew samples. For cold brew coffees, the extraction yield of antioxidant compounds decreased as degree of roast increased, suggesting that cold water is less effective than hot water at extracting the same suite of compounds. Moreover, a strong correlation was observed between total CQA concentration and TA with a Pearson correlation coefficient of 0.94 for cold brew coffees, while the same analysis reveals a weak correlation for the hot brew counterparts with a Pearson correlation coefficient of 0.21, in agreement with previous study by Rao and Fuller [10].

Results from previous studies on the TAC of roasted coffee have been somewhat inconclusive, and the discrepancies among those studies have been well documented [6,28,44,45]. Differences among various methods used to quantify the TAC of coffee brews have also been reported [6,34,37,38,39,42,46]. Catelani et al. highlighted that multiple factors contribute to the difficulty of directly comparing antioxidant activities from different studies [45]. Bean origin [47] and varietal [48], roasting procedure [42], and extraction methods [49,50] all contribute to a lack of consistency among TAC profiles. 

It is widely accepted that, as roasting occurs, phenolic compounds decrease in abundance while melanoidin compounds are produced. Previous studies have found that the increase in melanoidins serves to maintain the level of TAC in coffees as degree of roast increases [6,42,51]. Two previous studies by Vignoli et al. showed that degree of roast had little effect on the TAC of hot brew coffee [6,28], which agrees with the TAC profile observed in hot brew coffee here. Vignoli et al. argued that, although prolonged roasting may not produce enough additional antioxidant compounds to compensate for the loss of phenolic compounds, significant structural changes in the sold bean matrix may increase the extraction efficiency for all antioxidant compounds [6,28].

Little work has been published on the TAC of cold brew coffees as a function of roast level. Rao and Fuller found that, for a variety of regions, medium roast hot brew coffees had higher TAC than their cold brew counterparts, as evaluated using the ABTS decolorization assay [10]. Results from a recent study by Bilge generally found that hot brew French press coffee resulted in the highest antioxidant capacity across all roast levels, grind sizes, and brewing methods tested, using the 2,2-diphenyl-1-picrylhydrazyl (DPPH) decolorization assay [24]. This study serves to further elucidate the role of roasting in the production of antioxidant compounds in cold brew coffees. The increasing difference in TAC between hot and cold brew coffees with increasing degree of roast indicates that the chemical and physical changes that occur during roasting result in antioxidant compounds that are less available and less soluble during the cold brew process. Water extraction temperature influences the yield of antioxidant compounds across all roasts, but most significantly in dark roast coffees. Melanoidins and other antioxidant compounds produced during the roasting process may not be soluble in low temperature water, thus reducing the extraction efficiency of the cold brew process. 

### 3.4. Caffeine

The caffeine concentrations of all coffees analyzed were comparable regardless of roast level or brewing temperatures, consistent with previous studies [6,7,25,27,39,46,52,53,54,55]. However, a decrease in caffeine concentrations due to degradation during roasting has been observed in other studies [48,56]. Moreover, caffeine concentrations in cold brew samples were found to be similar to those in hot brew samples, in agreement with recent studies [7,9,57].

### 3.5. Roasting and Extraction Process

Complex structural changes to bean matrix porosity, in the presence of intra- and intergranular pore water, make the extraction process during brewing a dynamic interaction between solid and liquid phases. Mateus et al. found that closed porosity in roasted coffee grinds decreased from ~30% in dry coffee to ~5% in coffee with 30% w/w water content [58], indicating that the wetting process changes the porosity network of the grinds. This restructuring was largely due to polymer plasticization and swelling of the matrix when wetted, illustrating the importance of the water–coffee matrix interface during the extraction process [58]. The physical alterations of the solid bean matrix due to roasting are sensitive to degree of wetting, which is in turn affected by water extraction temperature. Moreover, the degradation of the coffee cellular matrix during the roasting process may also lead to resolubilization of celluloses, carbohydrates, and denatured proteins [27,29]. These factors influence the solubility and availability of organic compounds during the production of hot and cold brew coffees and likely contribute to the differential pH, TTA, and TAC of the resulting coffee beverages. 

Beyond these important physical changes, roasting causes significant chemical reactions that control a coffee’s aroma, taste, and final soluble yields. During roasting, CQA concentrations decrease due to transformation into chlorogenic acid lactones [17] and partial incorporation into the production of melanoidin compounds [59,60]. Additional compounds are also produced during roasting that are thought to contribute to the TAC of coffee, e.g., volatile heterocyclic compounds [61]. Beyond phenolic acid changes, the quantities of other important acidic compounds also vary as a function of degree of roast. Some organic acids, such as citric and malic acids, are present in green beans but decompose during the roasting process [62], while other acids are produced through a carbohydrate degradation pathway during roasting. These include acetic, formic, glycolic, and lactic acids [11].

## 4. Conclusions

This study found that pH, TTA, concentration of browned compounds, and TAC for both cold and hot brew coffees varied by each of the three roast levels tested. As the degree of roast increased, the differences in these measurements between cold and hot brew coffees also increased. The physical and chemical changes undergone by the solid bean matrix during the roasting process potentially altered mass transfer rates, solubility of various compounds, and extraction yield as a function of water temperature. 

The observed differences in pH, TTA, and TAC between cold and hot brew coffees were likely due, in part, to (1) the structural and chemical changes undergone by coffee beans during the roasting process and (2) the interaction of the solid bean matrix with extraction waters at varying temperatures. The hot brew coffee method used in this study, with an initial water contact temperature of 100 °C, is a high-energy system that results in a fast rate of soluble compound extraction [63]. It is possible that hot water wets the oily surface of the grinds more effectively than cold water, allowing for the extraction of more compounds. 

However, the similar CQA and caffeine concentrations in both hot and cold brew coffee suggest that wetting is not a dominant mechanism in the differences between hot and cold brew coffees. Rather, wetting may be more or less important depending on the polarity and molar mass of the compounds being extracted. The water extraction temperature difference in hot and cold brewing causes differential solubility of higher molecular mass compounds—specifically, melanoidins [33]. The extraction behavior and antioxidant activity of melanoidins in coffees have not been fully identified, as the compounds are often co-present in complex mixtures with polysaccharides, proteins, and other phenolic compounds [60]. The work presented here suggests that water extraction temperature influences wetting and solid bean matrix behavior, as well as compound solubility and extraction potential. The temperature-dependence of solubility, specifically for melanoidin compounds, is likely the cause of the differences in pH, TTA, and TAC between the hot and cold brew coffees studied here.

## Figures and Tables

**Table 1:** Physicochemical characteristics of cold and hot brew coffee samples at three different degrees of roast. TTA: total titratable acids to a pH 6.5 expressed in ml of 0.1 N NaOH per 40 mL of coffee, TDS: total dissolved solids, TAC: total antioxidant capacity determined by the ABTS decolorization assay expressed in mmol of Trolox per liter of coffee.

Roasted Samples	pH	TTA (mL of 0.1 N NaOH)	TDS (%)	Browned Compounds (Abs_420_)	Caffeine (mg/L)	Total CQA (mg/L)	TAC (mmol Trolox/L Coffee)
**Cold Brew**							
**R_1_**	5.00 ± 0.08 ^a,A^	4.66 ± 0.05 ^a,A^	1.88 ± 0.06 ^a,A^	0.358 ± 0.004 ^a,A^	1114 ± 56 ^a,A^	1535 ± 28 ^a,A^	13.09 ± 0.22 ^a,A^
**R_2_**	5.30 ± 0.01 ^b,A^	3.15 ± 0.06 ^b,A^	2.06 ± 0.04 ^b,A^	0.386 ± 0.006 ^b,A^	1036 ± 19 ^b,A^	1733 ± 11 ^b,A^	11.11 ± 0.33 ^b,A^
**R_3_**	5.75 ± 0.02 ^c,A^	1.53 ± 0.06 ^c,A^	2.05 ± 0.05 ^b,A^	0.422 ± 0.015 ^c,A^	1962 ± 41 ^c,A^	1308 ± 10 ^c,A^	10.13 ± 0.59 ^c,A^
**Hot Brew**							
**R_1_**	4.80 ± 0.01 ^a,B^	4.82 ± 0.13 ^a,B^	1.96 ± 0.05 ^a,B^	0.456 ± 0.018 ^a,B^	1095 ± 65 ^a,A^	1536 ± 46 ^a,A^	13.89 ± 0.24 ^a,B^
**R_2_**	5.04 ± 0.01 ^b,B^	3.55 ± 0.14 ^b,B^	2.12 ± 0.04 ^b,A^	0.572 ± 0.004 ^b,B^	1056 ± 47 ^a,A^	1719 ± 33 ^b,A^	13.99 ± 0.44 ^a,B^
**R_3_**	5.39 ± 0.03 ^c,B^	2.06 ± 0.06 ^c,B^	2.23 ± 0.04 ^c,B^	0.777 ± 0.018 ^c,B^	1035 ± 39 ^a,A^	1301 ± 11 ^c,A^	13.60 ± 0.50 ^a,B^

Each value is the mean ± SD. Sample size is *n* = 9 for TDS, Caffeine, Total CQA, and TAC; *n* = 6 for Browned Compounds; and *n* = 3 for pH and TTA. The superscripts *a–c* denote significant (*p* < 0.05) differences among degrees of roast within the same brewing method as determined by the Tukey HDS post-tests. The superscripts *A* and *B* denote significant differences between cold and hot brewing methods at the same degree of roast.

**Table 2:** Total CQA and CQA isomer concentration of cold and hot brew samples at different degrees of roast.

Roasted Samples	Total CQA (mg/L)	5-CQA (mg/L)	4-CQA (mg/L)	3-CQA (mg/L)
**Cold Brew**				
**R_1_**	1535 ± 28 ^a,A^	757 ± 27 ^a,A^	419 ± 12 ^a,A^	359 ± 14 ^a,A^
**R_2_**	1733 ± 11 ^b,A^	353 ± 15 ^b,A^	210 ± 13 ^b,A^	170 ± 13 ^b,A^
**R_3_**	1308 ± 10 ^c,A^	147 ± 14 ^c,A^	188 ± 14 ^c,A^	173 ± 14 ^c,A^
**Hot Brew**				
**R_1_**	1536 ± 46 ^a,A^	787 ± 20 ^a,B^	413 ± 20 ^a,A^	336 ± 9 ^a,B^
**R_2_**	1719 ± 33 ^b,A^	350 ± 15 ^b,A^	204 ± 19 ^b,A^	165 ± 9 ^b,A^
**R_3_**	1301 ± 11 ^c,A^	144 ± 16 ^c,A^	186 ± 13 ^c,A^	171 ± 3 ^c,A^

Each value is the mean ± SD. *n* = 9. The superscripts *a–c* denote significant (*p* < 0.05) differences among degrees of roast within the same brewing method as determined by the Tukey HDS post-tests. The superscripts *A* and *B* denote significant differences between cold and hot brewing methods at the same degree of roast.

## Supplementary Materials

The following are available online at https://www.mdpi.com/2304-8158/9/7/902/s1. Table S1: ANOVA Summary Table for pH, Table S2: ANOVA Summary Table for Total Titratable Acidity (TTA), Table S3: ANOVA Summary Table for Total Dissolved Solids (TDS), Table S4: ANOVA Summary Table for Browned Compounds, Table S5: ANOVA Summary Table for Caffeine, Table S6: ANOVA Summary Table for Total CQA, Table S7: ANOVA Summary Table for Total Antioxidant Activities (TAC), Table S8: ANOVA Summary Table for 5-CQA, Table S9: ANOVA Summary Table for 4-CQA, Table S10: ANOVA Summary Table for 3-CQA.
