# Supplementary material for: Physiochemical Characteristics of Hot and Cold Brew Coffee Chemistry: The Effects of Roast Level and Brewing Temperature on Compound Extraction
Source: Foods. 2020 Jul 9;9(7):902. doi: 10.3390/foods9070902 (PMC7404565; doi:10.3390/foods9070902)
Supplement: Supplementary file 1 [file foods-09-00902-s001.pdf]

Table S1. ANOVA Summary Table for pH

Source of Variation	*SS*	*df*	*MS*	*F*	*P-value*
**Degrees of Roast**	1.376178	2	0.688089	604.1756	9.04E-13
**Brewing Methods**	0.330756	1	0.330756	290.4195	8.96E-10
**Interaction**	0.019911	2	0.009956	8.741463	0.004546
**Within**	0.013667	12	0.001139		
**Total**	**1.740511111**	**17**			

Table S2. ANOVA Summary Table for Total Titratable Acidity (TTA)

Source of Variation	*SS*	*df*	*MS*	*F*	*P-value*
**Degrees of Roast**	26.01741	2	13.00871	1558.966	3.18E-15
**Brewing Methods**	0.597689	1	0.597689	71.62716	2.1E-06
**Interaction**	0.106411	2	0.053206	6.376165	0.012983
**Within**	0.100133	12	0.008344		
**Total**	**26.82164**	**17**			

Table S3. ANOVA Summary Table for Total Dissolved Solids (TDS)

Source of Variation	*SS*	*df*	*MS*	*F*	*P-value*
**Degrees of Roast**	0.46283033	2	0.23141517	101.6	5.6146E-18
**Brewing Methods**	0.1526415	1	0.1526415	67.0153846	1.1534E-10
**Interaction**	0.041209	2	0.0206045	9.04615385	0.00046363
**Within**	0.10933	48	0.00227771		
**Total**	**0.76601083**	**53**			

Table S4. ANOVA Summary Table for Browned Compounds

Source of Variation	*SS*	*df*	*MS*	*F*	*P-value*
**Degrees of Roast**	0.226833	2	0.113417	710.5567	5.39E-26

**Brewing Methods**	0.40896	1	0.40896	2562.14	1.3E-30
**Interaction**	0.10312	2	0.05156	323.0244	5.1E-21
**Within**	0.004789	30	0.00016		
**Total**	**0.743702**	**35**			

Table S5. ANOVA Summary Table for Caffeine

Source of Variation	*SS*	*df*	*MS*	*F*	*P-value*
**Degrees of Roast**	101364.8	2	50682.39	23.22708	8.8E-08
**Brewing Methods**	8346.329	1	8346.329	3.825014	0.056327
**Interaction**	19211.84	2	9605.921	4.402269	0.017563
**Within**	104737.9	48	2182.039		
**Total**	**233660.8**	**53**			

Table S6. ANOVA Summary Table for Total CQA

Source of Variation	*SS*	*df*	*MS*	*F*	*P-value*
**Degrees of Roast**	14095067	2	7047533	9660.225	2.88E-63
**Brewing Methods**	666.7089	1	666.7089	0.913874	0.343879
**Interaction**	525.3809	2	262.6905	0.360076	0.699491
**Within**	35017.98	48	729.5413		
**Total**	**14131277**	**53**			

Table S7. ANOVA Summary Table for Total Antioxidant Activities (TAC)

Source of Variation	*SS*	*df*	*MS*	*F*	*P-value*
**Degrees of Roast**	23.9262	2	11.9631	71.3637	4.17E-15
**Brewing Methods**	76.70594	1	76.70594	457.5753	3.46E-26
**Interaction**	17.7527	2	8.876348	52.95023	7.18E-13

**Within**	8.046512	48	0.167636		
**Total**	**126.4314**	**53**			

Table S8. ANOVA Summary Table for 5-CQA

Source of Variation	*SS*	*df*	*MS*	*F*	*P-value*
**Degrees of Roast**	3673887.15	2	1836943.57	7788.64327	4.9879E-61
**Brewing Methods**	864.010993	1	864.010993	3.66340779	0.06159219
**Interaction**	3293.52552	2	1646.76276	6.98227635	0.00217947
**Within**	11320.7511	48	235.84898		
**Total**	**126.4314**	**53**			

Table S9. ANOVA Summary Table for 4-CQA

Source of Variation	*SS*	*df*	*MS*	*F*	*P-value*
**Degrees of Roast**	997385.698	2	498692.849	4656.05037	1.0941E-55
**Brewing Methods**	305.594701	1	305.594701	2.85318773	0.09767962
**Interaction**	50.9817054	2	25.4908527	0.23799558	0.78913073
**Within**	5141.10778	48	107.106412		
**Total**	**1002883.38**	**53**			

Table S10. ANOVA Summary Table for 3-CQA

Source of Variation	*SS*	*df*	*MS*	*F*	*P-value*
**Degrees of Roast**	704752.386	2	352376.193	5421.97688	2.8791E-57
**Brewing Methods**	1423.81735	1	1423.81735	21.9081337	2.3702E-05
**Interaction**	1162.14942	2	581.07471	8.94093784	0.0005005
**Within**	3119.53696	48	64.9903534		
**Total**	**710457.89**	**53**			
